# (2-(Benzo[*d*]thia­zol-2yl-meth­oxy)-5-chloro­phen­yl)(phen­yl)methanone

**DOI:** 10.1107/S1600536812041888

**Published:** 2012-10-13

**Authors:** K. N. Venugopala, Susanta K. Nayak, Thavendran Govender, Hendrik G. Kruger, Glenn E. M. Maguire

**Affiliations:** a School of Pharmacy and Pharmacology, University of KwaZulu-Natal, Durban 4000, South Africa; b Center for Nano Science and Technology@Polimi, Istituto Italiano di Tecnologia, Via Pascoli 70/3-20133 Milan, Italy; c School of Chemistry and Physics, University of KwaZulu-Natal, Durban 4000, South Africa

## Abstract

In the title compound, C_21_H_14_ClNO_2_S, the dihedral angle between the benzothia­zole and diphenyl methanone groups is 68.6 (2)°. The crystal structure consists of dimeric units generated by C—H⋯N bonds, further linked by C—H⋯O bonds and C—H⋯π and π–π inter­actions [centroid–centroiddistance = 3.856 (2) Å], which lead to a criss-cross assembly parallel to (001).

## Related literature

For background to the applications of benzothia­zole derivatives, see: Rana *et al.* (2007 ▶); Telvekar *et al.* (2012 ▶); Saeed *et al.* (2010 ▶).
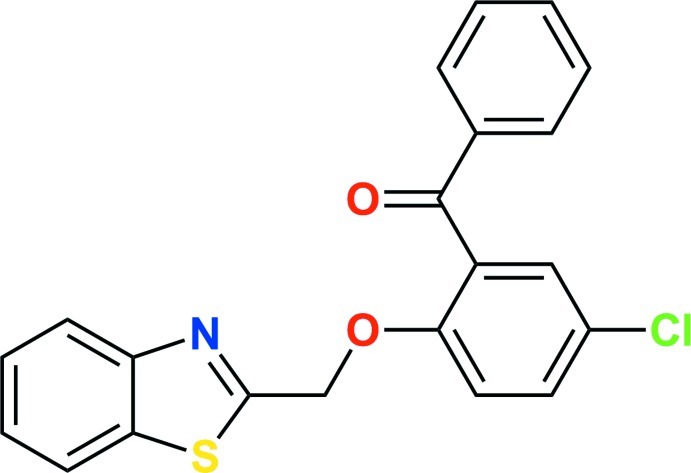


## Experimental

### 

#### Crystal data

C_21_H_14_ClNO_2_S
*M* *_r_* = 379.84
Orthorhombic, 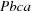

*a* = 7.4598 (3) Å
*b* = 19.3131 (8) Å
*c* = 24.4002 (9) Å
*V* = 3515.4 (2) Å^3^
*Z* = 8
Mo *K*α radiation
μ = 0.35 mm^−1^
*T* = 173 K
0.22 × 0.16 × 0.03 mm

#### Data collection

Nonius KappaCCD diffractometer
Absorption correction: multi-scan (*SADABS*; Sheldrick, 2008 ▶) *T*_min_ = 0.927, *T*_max_ = 0.990
52834 measured reflections
3206 independent reflections
2576 reflections with *I* > 2σ(*I*)
*R*_int_ = 0.063

#### Refinement

*R*[*F* ^2^ > 2σ(*F* ^2^)] = 0.059
*wR*(*F* ^2^) = 0.200
*S* = 1.30
3206 reflections
235 parameters
H-atom parameters constrained
Δρ_max_ = 0.66 e Å^−3^
Δρ_min_ = −0.33 e Å^−3^

### 

Data collection: *COLLECT* (Nonius, 1998 ▶); cell refinement: *DENZO-SMN* (Otwinowski & Minor, 1997 ▶); data reduction: *DENZO-SMN*; program(s) used to solve structure: *SHELXS97* (Sheldrick, 2008 ▶); program(s) used to refine structure: *SHELXL97* (Sheldrick, 2008 ▶); molecular graphics: *ORTEP-3 for Windows* (Farrugia, 1997 ▶) and *Mercury* (Macrae *et al.*, 2008 ▶); software used to prepare material for publication: *PLATON* (Spek, 2009 ▶) and *PARST* (Nardelli, 1995 ▶).

## Supplementary Material

Crystal structure: contains datablock(s) global, I. DOI: 10.1107/S1600536812041888/bg2479sup1.cif

Structure factors: contains datablock(s) I. DOI: 10.1107/S1600536812041888/bg2479Isup2.hkl

Supplementary material file. DOI: 10.1107/S1600536812041888/bg2479Isup3.cml

Additional supplementary materials:  crystallographic information; 3D view; checkCIF report

## Figures and Tables

**Table 1:** Hydrogen-bond geometry (Å, °) *Cg*1 is the centroid of the S1/C1/C6/N1/C7 ring.

*D*—H⋯*A*	*D*—H	H⋯*A*	*D*⋯*A*	*D*—H⋯*A*
C17—H17⋯N1^i^	0.95	2.56	3.432 (5)	153
C5—H5⋯O2^ii^	0.95	2.59	3.478 (5)	155
C18—H18⋯*Cg*1^iii^	0.95	2.62	3.433 (5)	144

Symmetry codes: (i) 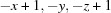; (ii) 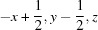; (iii) 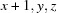.

**Fig. 1.**
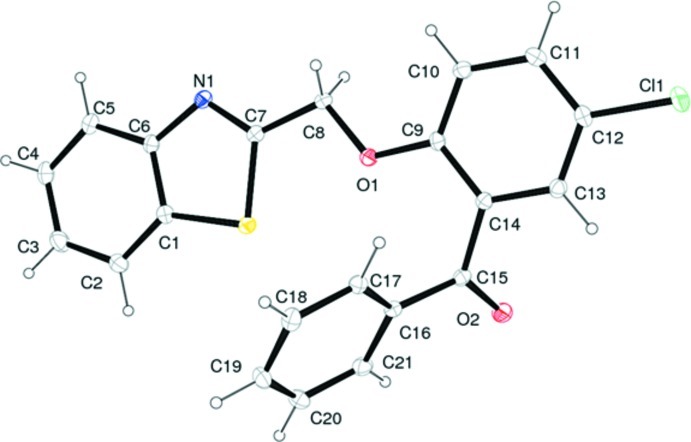
Molecular structure of the title compound showing the atom labelling scheme with displacement ellipsoids for non-H atoms at 50% probability level.

**Fig. 2.**
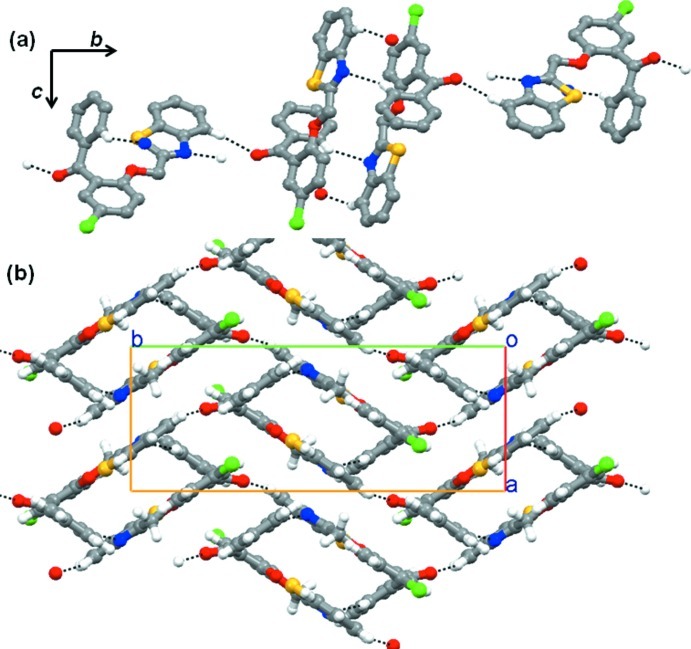
Packing views of the title compound. (a) Showing in detail the formation of dimers (C—H···N bonds) and their interaction (C—H···O bonds. (b). The criss-cross molecular assembly perpendicular to the c axis.

## supplementary crystallographic information

### Comment

Our interest in the molecular design of the benzothiazole moiety is due to its diverse chemical and biological activities. Benzothiazole derivatives exhibit biological activities as antitumor, antiinflammatory, analgesic, antimicrobial, and potential anti HIV agents *etc*. Still the variety of biological features of new benzothiazole derivatives are of great scientific interest Rana *et al.* (2007); Telvekar *et al.* (2012); Saeed *et al.* (2010). Here, we report the single-crystal structure of the title compound, C_21_H_14_ClNO_2_S.

The molecule adopts a conformation with a 68.6 (2)° dihedral angle between the planes of the benzothiazole and diphenyl methanone groups (Fig. 1). The weak C—H···N hydrogen bonds (Table 1, 1st entry) form dimeric units (Fig. 2a), in turn linked by the C—H···O ones (Table 1, 2nd entry) into planar arrays parallel to (001). This 2D structure is reinforced by a C-H···π bond involving the five-membered ring S1/C1/C6/N1/C7 (centroid Cg1) (Table 1, third entry) and a π···π bond between the C10—C14 six-membered ring (centroid Cg2) and the C16—C21 six-membered ring (centroid Cg3), viz., Cg2···Cg3^i^, (i): 1/2 + *x*, 1/2 - *y*, -*z*, with an intercentroid distance of 3.856 (2) Å, all these interactions leading to the criss-cross assembly depicted in Fig 2b.

### Experimental

To a solution of 2-(chloromethyl)-benzo-thiazole (0.5 g, 0.0027 mol) and (5-chloro-2-hydroxyphenyl)(phenyl)methanone (0.0027 mol) in dry THF, dry potassium carbonate (0.38 g, 0.0027 mol) was added and stirred at room temperature. The reaction mixture was concentrated to remove the solvent, diluted with ethyl acetate, washed with water, brine solution and dried over anhydrous sodium sulfate. The organic layer was concentrated to yield a residue which was purified by column chromatography using ethyl acetate and n-hexane as eluent (7:3, Rf = 0.68) to afford as a white solid product (Yield 70.8%; m. p. 403 (2) K).Suitable crystals for single-crystal X-ray study were obtained by slow evaporation crystallization from ethanol solvent at room temperature.

### Refinement

All H atoms were positioned geometrically and refined using a riding model with *U*_iso_(H)= 1.2 *U*_eq_(C). The space group might be considered as Pbca only on average, since there are many violations of the a glide condition. This may be the reason for some Fo-Fc discrepancies. Refinement in lower symmetry groups, however, did not improve the results.

### Crystal data

**Table:**

C_21_H_14_ClNO_2_S	*D*_x_ = 1.435 Mg m^−^^3^
*M**_r_* = 379.84	Melting point: 403(2) K
Orthorhombic, *P**b**c**a*	Mo *K*α radiation, λ = 0.71073 Å
Hall symbol: -P 2ac 2ab	Cell parameters from 560 reflections
*a* = 7.4598 (3) Å	θ = 1.7–25.3°
*b* = 19.3131 (8) Å	µ = 0.35 mm^−^^1^
*c* = 24.4002 (9) Å	*T* = 173 K
*V* = 3515.4 (2) Å^3^	Plate, colourless
*Z* = 8	0.22 × 0.16 × 0.03 mm
*F*(000) = 1568	

### Data collection

**Table:**

Nonius KappaCCD diffractometer	3206 independent reflections
Radiation source: fine-focus sealed tube	2576 reflections with *I* > 2σ(*I*)
Graphite monochromator	*R*_int_ = 0.063
1.2° φ scans and ω scans	θ_max_ = 25.3°, θ_min_ = 2.3°
Absorption correction: multi-scan (*SADABS*; Sheldrick, 2008)	*h* = −8→8
*T*_min_ = 0.927, *T*_max_ = 0.990	*k* = −23→23
52834 measured reflections	*l* = −29→29

### Refinement

**Table:**

Refinement on *F*^2^	Primary atom site location: structure-invariant direct methods
Least-squares matrix: full	Secondary atom site location: difference Fourier map
*R*[*F*^2^ > 2σ(*F*^2^)] = 0.059	Hydrogen site location: inferred from neighbouring sites
*wR*(*F*^2^) = 0.200	H-atom parameters constrained
*S* = 1.30	*w* = 1/[σ^2^(*F*_o_^2^) + (0.071*P*)^2^ + 8.1626*P*] where *P* = (*F*_o_^2^ + 2*F*_c_^2^)/3
3206 reflections	(Δ/σ)_max_ < 0.001
235 parameters	Δρ_max_ = 0.66 e Å^−^^3^
0 restraints	Δρ_min_ = −0.33 e Å^−^^3^

### Special details

**Table:**

Geometry. All esds (except the esd in the dihedral angle between two l.s. planes) are estimated using the full covariance matrix. The cell esds are taken into account individually in the estimation of esds in distances, angles and torsion angles; correlations between esds in cell parameters are only used when they are defined by crystal symmetry. An approximate (isotropic) treatment of cell esds is used for estimating esds involving l.s. planes.
Refinement. Refinement of F^2^ against ALL reflections. The weighted R-factor wR and goodness of fit S are based on F^2^, conventional R-factors R are based on F, with F set to zero for negative F^2^. The threshold expression of F^2^ > 2sigma(F^2^) is used only for calculating R-factors(gt) etc. and is not relevant to the choice of reflections for refinement. R-factors based on F^2^ are statistically about twice as large as those based on F, and R- factors based on ALL data will be even larger.

### Fractional atomic coordinates and isotropic or equivalent isotropic displacement parameters (Å^2^)

**Table:**

	*x*	*y*	*z*	*U*_iso_*/*U*_eq_	
S1	0.34601 (15)	0.06802 (6)	0.39552 (4)	0.0309 (3)	
Cl1	0.68528 (18)	0.27003 (7)	0.68940 (5)	0.0470 (4)	
O1	0.4110 (4)	0.11200 (15)	0.50149 (11)	0.0315 (7)	
N1	0.1614 (5)	−0.02826 (17)	0.44408 (14)	0.0273 (8)	
C6	0.1481 (5)	−0.0405 (2)	0.38812 (17)	0.0270 (9)	
C7	0.2577 (5)	0.0259 (2)	0.45299 (16)	0.0256 (9)	
C8	0.2953 (6)	0.0542 (2)	0.50881 (16)	0.0276 (9)	
H8A	0.1825	0.0688	0.5268	0.033*	
H8B	0.3537	0.0187	0.5319	0.033*	
O2	0.5685 (4)	0.29934 (15)	0.47346 (12)	0.0373 (8)	
C14	0.5686 (5)	0.2081 (2)	0.53676 (16)	0.0245 (9)	
C15	0.5962 (5)	0.2376 (2)	0.48050 (17)	0.0258 (9)	
C16	0.6632 (5)	0.1936 (2)	0.43523 (16)	0.0240 (9)	
C17	0.7650 (5)	0.1344 (2)	0.44537 (16)	0.0266 (9)	
H17	0.7872	0.1203	0.4820	0.032*	
C13	0.6296 (5)	0.2462 (2)	0.58122 (17)	0.0286 (9)	
H13	0.6917	0.2885	0.5753	0.034*	
C9	0.4719 (5)	0.1466 (2)	0.54683 (16)	0.0252 (9)	
C19	0.8018 (7)	0.1161 (3)	0.34894 (19)	0.0400 (11)	
H19	0.8501	0.0899	0.3195	0.048*	
C10	0.4403 (6)	0.1248 (2)	0.60021 (17)	0.0305 (9)	
H10	0.3735	0.0837	0.6068	0.037*	
C18	0.8338 (6)	0.0963 (2)	0.40225 (19)	0.0340 (10)	
H18	0.9036	0.0562	0.4095	0.041*	
C11	0.5061 (6)	0.1629 (2)	0.64375 (17)	0.0336 (10)	
H11	0.4862	0.1477	0.6803	0.040*	
C1	0.2398 (6)	0.0070 (2)	0.35491 (17)	0.0286 (9)	
C12	0.6004 (6)	0.2229 (2)	0.63404 (17)	0.0301 (10)	
C20	0.6980 (7)	0.1747 (3)	0.33833 (18)	0.0409 (12)	
H20	0.6730	0.1878	0.3016	0.049*	
C5	0.0510 (6)	−0.0945 (2)	0.36427 (19)	0.0347 (10)	
H5	−0.0113	−0.1271	0.3863	0.042*	
C4	0.0477 (7)	−0.0993 (2)	0.30759 (19)	0.0407 (11)	
H4	−0.0177	−0.1357	0.2907	0.049*	
C21	0.6322 (6)	0.2134 (2)	0.38092 (18)	0.0340 (10)	
H21	0.5647	0.2540	0.3735	0.041*	
C2	0.2347 (7)	0.0017 (3)	0.29783 (19)	0.0400 (11)	
H2	0.2961	0.0341	0.2754	0.048*	
C3	0.1385 (7)	−0.0517 (3)	0.2751 (2)	0.0442 (12)	
H3	0.1340	−0.0562	0.2364	0.053*	

### Atomic displacement parameters (Å^2^)

**Table:**

	*U*^11^	*U*^22^	*U*^33^	*U*^12^	*U*^13^	*U*^23^
S1	0.0343 (6)	0.0302 (6)	0.0282 (6)	−0.0080 (5)	0.0014 (4)	0.0005 (4)
Cl1	0.0576 (8)	0.0537 (8)	0.0296 (6)	−0.0104 (6)	−0.0051 (5)	−0.0114 (5)
O1	0.0351 (17)	0.0341 (16)	0.0253 (14)	−0.0140 (14)	0.0015 (12)	−0.0025 (12)
N1	0.0278 (19)	0.0235 (18)	0.0308 (18)	0.0005 (15)	−0.0023 (15)	0.0018 (15)
C6	0.023 (2)	0.026 (2)	0.032 (2)	0.0039 (17)	−0.0003 (17)	−0.0032 (17)
C7	0.021 (2)	0.025 (2)	0.031 (2)	0.0018 (17)	0.0013 (17)	0.0008 (17)
C8	0.028 (2)	0.026 (2)	0.029 (2)	−0.0050 (18)	0.0022 (17)	0.0020 (17)
O2	0.051 (2)	0.0240 (16)	0.0366 (17)	0.0032 (14)	0.0062 (15)	0.0024 (13)
C14	0.023 (2)	0.024 (2)	0.027 (2)	0.0019 (17)	0.0009 (17)	−0.0010 (16)
C15	0.022 (2)	0.025 (2)	0.030 (2)	−0.0031 (17)	−0.0016 (17)	0.0019 (17)
C16	0.023 (2)	0.023 (2)	0.026 (2)	−0.0022 (17)	0.0002 (16)	0.0027 (17)
C17	0.024 (2)	0.028 (2)	0.027 (2)	−0.0004 (18)	−0.0004 (17)	0.0006 (17)
C13	0.026 (2)	0.028 (2)	0.032 (2)	−0.0024 (18)	0.0015 (18)	−0.0033 (17)
C9	0.023 (2)	0.028 (2)	0.025 (2)	0.0003 (17)	−0.0020 (16)	−0.0040 (16)
C19	0.044 (3)	0.043 (3)	0.033 (2)	−0.005 (2)	0.011 (2)	−0.011 (2)
C10	0.030 (2)	0.031 (2)	0.030 (2)	−0.0041 (19)	0.0025 (18)	0.0026 (18)
C18	0.026 (2)	0.032 (2)	0.044 (3)	0.0014 (18)	0.0013 (19)	−0.007 (2)
C11	0.036 (2)	0.041 (3)	0.024 (2)	0.001 (2)	0.0025 (18)	0.0024 (19)
C1	0.025 (2)	0.031 (2)	0.030 (2)	0.0018 (18)	−0.0015 (18)	−0.0024 (18)
C12	0.029 (2)	0.035 (2)	0.026 (2)	0.0031 (19)	−0.0021 (17)	−0.0066 (18)
C20	0.056 (3)	0.044 (3)	0.023 (2)	−0.005 (2)	0.000 (2)	0.004 (2)
C5	0.033 (2)	0.028 (2)	0.043 (3)	−0.001 (2)	−0.002 (2)	−0.005 (2)
C4	0.044 (3)	0.038 (3)	0.040 (3)	−0.004 (2)	−0.008 (2)	−0.014 (2)
C21	0.043 (3)	0.029 (2)	0.030 (2)	−0.002 (2)	−0.004 (2)	0.0076 (18)
C2	0.046 (3)	0.043 (3)	0.030 (2)	−0.006 (2)	0.003 (2)	−0.001 (2)
C3	0.049 (3)	0.053 (3)	0.031 (2)	−0.004 (2)	−0.002 (2)	−0.008 (2)

### Geometric parameters (Å, º)

**Table:**

S1—C1	1.731 (4)	C13—H13	0.9500
S1—C7	1.750 (4)	C9—C10	1.389 (6)
Cl1—C12	1.747 (4)	C19—C18	1.377 (7)
O1—C9	1.370 (5)	C19—C20	1.396 (7)
O1—C8	1.422 (5)	C19—H19	0.9500
N1—C7	1.288 (5)	C10—C11	1.383 (6)
N1—C6	1.389 (5)	C10—H10	0.9500
C6—C5	1.397 (6)	C18—H18	0.9500
C6—C1	1.402 (6)	C11—C12	1.377 (6)
C7—C8	1.494 (6)	C11—H11	0.9500
C8—H8A	0.9900	C1—C2	1.397 (6)
C8—H8B	0.9900	C20—C21	1.371 (6)
O2—C15	1.223 (5)	C20—H20	0.9500
C14—C13	1.388 (6)	C5—C4	1.386 (6)
C14—C9	1.411 (6)	C5—H5	0.9500
C14—C15	1.500 (6)	C4—C3	1.390 (7)
C15—C16	1.480 (6)	C4—H4	0.9500
C16—C17	1.394 (6)	C21—H21	0.9500
C16—C21	1.399 (6)	C2—C3	1.372 (7)
C17—C18	1.383 (6)	C2—H2	0.9500
C17—H17	0.9500	C3—H3	0.9500
C13—C12	1.382 (6)		
			
C1—S1—C7	88.3 (2)	C20—C19—H19	120.1
C9—O1—C8	118.8 (3)	C11—C10—C9	119.9 (4)
C7—N1—C6	110.1 (3)	C11—C10—H10	120.0
N1—C6—C5	125.0 (4)	C9—C10—H10	120.0
N1—C6—C1	115.0 (4)	C19—C18—C17	120.5 (4)
C5—C6—C1	120.0 (4)	C19—C18—H18	119.8
N1—C7—C8	123.8 (4)	C17—C18—H18	119.8
N1—C7—S1	116.9 (3)	C12—C11—C10	119.9 (4)
C8—C7—S1	119.3 (3)	C12—C11—H11	120.1
O1—C8—C7	106.6 (3)	C10—C11—H11	120.1
O1—C8—H8A	110.4	C2—C1—C6	121.0 (4)
C7—C8—H8A	110.4	C2—C1—S1	129.3 (4)
O1—C8—H8B	110.4	C6—C1—S1	109.7 (3)
C7—C8—H8B	110.4	C11—C12—C13	121.0 (4)
H8A—C8—H8B	108.6	C11—C12—Cl1	119.4 (3)
C13—C14—C9	118.6 (4)	C13—C12—Cl1	119.6 (3)
C13—C14—C15	118.0 (4)	C21—C20—C19	120.0 (4)
C9—C14—C15	123.3 (4)	C21—C20—H20	120.0
O2—C15—C16	120.8 (4)	C19—C20—H20	120.0
O2—C15—C14	118.4 (4)	C4—C5—C6	118.3 (4)
C16—C15—C14	120.7 (3)	C4—C5—H5	120.8
C17—C16—C21	118.8 (4)	C6—C5—H5	120.8
C17—C16—C15	121.5 (4)	C5—C4—C3	121.1 (4)
C21—C16—C15	119.6 (4)	C5—C4—H4	119.5
C18—C17—C16	120.2 (4)	C3—C4—H4	119.5
C18—C17—H17	119.9	C20—C21—C16	120.7 (4)
C16—C17—H17	119.9	C20—C21—H21	119.7
C12—C13—C14	120.3 (4)	C16—C21—H21	119.7
C12—C13—H13	119.8	C3—C2—C1	118.2 (4)
C14—C13—H13	119.8	C3—C2—H2	120.9
O1—C9—C10	123.6 (4)	C1—C2—H2	120.9
O1—C9—C14	116.1 (3)	C2—C3—C4	121.4 (4)
C10—C9—C14	120.4 (4)	C2—C3—H3	119.3
C18—C19—C20	119.8 (4)	C4—C3—H3	119.3
C18—C19—H19	120.1		
			
C7—N1—C6—C5	−178.9 (4)	O1—C9—C10—C11	−179.8 (4)
C7—N1—C6—C1	0.2 (5)	C14—C9—C10—C11	−1.0 (6)
C6—N1—C7—C8	178.7 (4)	C20—C19—C18—C17	−0.5 (7)
C6—N1—C7—S1	−0.7 (5)	C16—C17—C18—C19	−0.4 (6)
C1—S1—C7—N1	0.8 (3)	C9—C10—C11—C12	1.0 (7)
C1—S1—C7—C8	−178.6 (3)	N1—C6—C1—C2	−178.7 (4)
C9—O1—C8—C7	−177.9 (3)	C5—C6—C1—C2	0.5 (7)
N1—C7—C8—O1	177.7 (4)	N1—C6—C1—S1	0.4 (5)
S1—C7—C8—O1	−2.9 (5)	C5—C6—C1—S1	179.6 (3)
C13—C14—C15—O2	−42.2 (5)	C7—S1—C1—C2	178.3 (5)
C9—C14—C15—O2	132.7 (4)	C7—S1—C1—C6	−0.6 (3)
C13—C14—C15—C16	135.0 (4)	C10—C11—C12—C13	0.5 (7)
C9—C14—C15—C16	−50.1 (6)	C10—C11—C12—Cl1	−179.2 (3)
O2—C15—C16—C17	152.4 (4)	C14—C13—C12—C11	−2.0 (6)
C14—C15—C16—C17	−24.8 (6)	C14—C13—C12—Cl1	177.7 (3)
O2—C15—C16—C21	−24.3 (6)	C18—C19—C20—C21	1.6 (7)
C14—C15—C16—C21	158.5 (4)	N1—C6—C5—C4	178.8 (4)
C21—C16—C17—C18	0.1 (6)	C1—C6—C5—C4	−0.3 (6)
C15—C16—C17—C18	−176.6 (4)	C6—C5—C4—C3	0.0 (7)
C9—C14—C13—C12	2.0 (6)	C19—C20—C21—C16	−1.9 (7)
C15—C14—C13—C12	177.1 (4)	C17—C16—C21—C20	1.1 (7)
C8—O1—C9—C10	6.1 (6)	C15—C16—C21—C20	177.8 (4)
C8—O1—C9—C14	−172.8 (3)	C6—C1—C2—C3	−0.5 (7)
C13—C14—C9—O1	178.4 (3)	S1—C1—C2—C3	−179.3 (4)
C15—C14—C9—O1	3.6 (6)	C1—C2—C3—C4	0.2 (8)
C13—C14—C9—C10	−0.5 (6)	C5—C4—C3—C2	0.0 (8)
C15—C14—C9—C10	−175.3 (4)		

### Hydrogen-bond geometry (Å, º)

Cg1 is the centroid of the S1/C1/C6/N1/C7 ring.

**Table:**

*D*—H···*A*	*D*—H	H···*A*	*D*···*A*	*D*—H···*A*
C17—H17···N1^i^	0.95	2.56	3.432 (5)	153
C5—H5···O2^ii^	0.95	2.59	3.478 (5)	155
C18—H18···*Cg*1^iii^	0.95	2.62	3.433 (5)	144

Symmetry codes: (i) −*x*+1, −*y*, −*z*+1; (ii) −*x*+1/2, *y*−1/2, *z*; (iii) *x*+1, *y*, *z*.
